# Feasibility of 35-mm Balloon-Expandable Transcatheter Pulmonary Valve Implantation in Extra-Large Native Right Ventricular Outflow Tracts

**DOI:** 10.1016/j.jaccas.2026.108879

**Published:** 2026-06-25

**Authors:** Abdullah Mohammed Al Farqani, Madan Mohan Maddali, Khalid Al Alawi, Umaima Khamis Al-Ghafri, Asim Al Balushi, Hamood Nasar Al-Kindi, Salim Nasser Al Maskari

**Affiliations:** a Department of Pediatric Cardiology, National Heart Center, The Royal Hospital, Muscat, Oman; b Department of Cardiac Anesthesia, National Heart Center, The Royal Hospital, Muscat, Oman; c Anesthesia and Intensive Care Residency Program, Oman Medical Specialty Board, Muscat, Oman; d Department of Cardiothoracic Surgery, National Heart Center, The Royal Hospital, Muscat, Oman

**Keywords:** bioprosthesis, cardiac catheterization, heart valve prosthesis implantation, pulmonary valve, pulmonary valve insufficiency, right ventricular outflow tract, tetralogy of Fallot, treatment outcome

## Abstract

**Background:**

Transcatheter pulmonary valve implantation is established for right ventricular outflow tract dysfunction but remains limited in extra-large native outflow tracts because of device size constraints.

**Case Summary:**

This case series describes implantation of a custom 35-mm balloon-expandable Myval valve in 4 patients with severe pulmonary regurgitation and right ventricular dilation who were unsuitable for conventional transcatheter therapy. Multimodality imaging and balloon interrogation consistently identified a functional landing zone of approximately 32 mm, guiding valve selection. Prestenting was applied in aneurysmal outflow tracts, while direct implantation was feasible in the presence of focal narrowing. Procedural success was achieved in all cases, with elimination of pulmonary regurgitation, low gradients, and no major complications. These findings demonstrate feasibility and reproducibility of this approach and support dynamic sizing to expand transcatheter options in extra-large native outflow tracts.

**Take-Home Message:**

Implantation of a custom 35-mm balloon-expandable valve in extra-large native right ventricular outflow tracts is feasible and reproducible, demonstrating that dynamic sizing and tailored procedural strategy can safely expand transcatheter pulmonary valve implantation to patients previously excluded by device size limitations.

## Visual Summary

**Figure:**
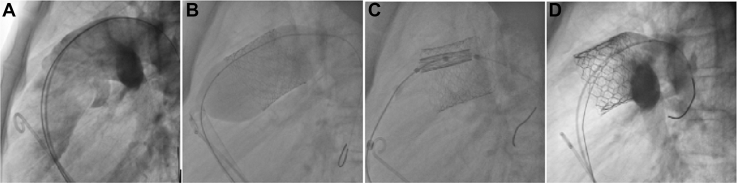

**Visual Summary:**
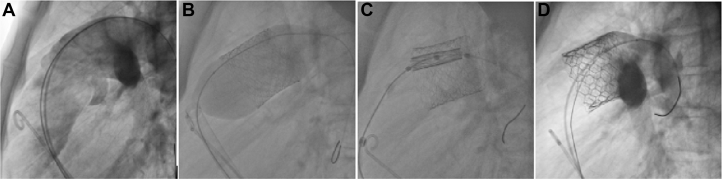
Transcatheter Pulmonary Valve Implantation Using a Custom 35-mm Balloon-Expandable Valve in Extra-Large Native Right Ventricular Outflow Tract Dynamic balloon interrogation consistently identified a functional landing zone of ∼32 mm despite markedly dilated native RVOT dimensions on static imaging. Prestenting was applied in aneurysmal outflow tracts to create a stable landing zone, whereas direct implantation was feasible in focal narrowing. Deployment of the 35-mm Myval valve achieved elimination of pulmonary regurgitation, low gradients, and stable anchoring without major complications. This series demonstrates the feasibility and reproducibility of large-diameter balloon-expandable transcatheter pulmonary valve implantation, expanding therapeutic options for patients previously excluded from transcatheter therapy. (A) RVOT angiogram demonstrated a markedly dilated native RVOT and main pulmonary artery, exceeding conventional transcatheter valve size limits. (B) Stenting of the RVOT was performed to create a suitable landing zone using 2 Optimus cobalt-chromium XXL 57-mm stents (AndraTec) deployed over a 40 × 40 mm Z-MED percutaneous transluminal valvuloplasty catheter (NuMED). (C) Introduction of the 35-mm Myval valve into the prepared landing zone was achieved using the Myval delivery system (Navigator delivery catheter, Meril Life Sciences). (D) A 35-mm balloon-expandable valve was deployed under rapid right ventricular pacing, resulting in stable positioning, complete elimination of pulmonary regurgitation, and low residual gradients. This staged illustration emphasizes the discrepancy between anatomical and functional dimensions and the role of dynamic sizing and tailored procedural strategy in enabling transcatheter pulmonary valve implantation in extra-large native outflow tracts. RVOT = right ventricular outflow tract.

Transcatheter pulmonary valve implantation (TPVI) has emerged as an established alternative to surgical intervention for right ventricular outflow tract (RVOT) dysfunction.^1^, ^2^, ^3^, ^4^ Despite its success, patients with markedly dilated native RVOTs remain excluded from current device options because of size limitations. Balloon-expandable systems are limited to diameters ≤29 mm, whereas self-expanding platforms rely on specific anatomical configurations for secure anchoring.^5^^,^^6^ Reports of larger balloon-expandable valves are limited to isolated experiences, leaving a significant gap in therapeutic strategies for this subgroup.^7^ Addressing this unmet need, we present to our knowledge the first case series evaluating the feasibility and procedural approach of a custom 35-mm balloon-expandable valve in extra-large native RVOTs, highlighting technical considerations and clinical implications for expanding TPVI candidacy.

**Take-Home Message**

• Implantation of a custom 35-mm balloon-expandable valve in extra-large native right ventricular outflow tracts is feasible and reproducible, demonstrating that dynamic sizing and tailored procedural strategy can safely expand transcatheter pulmonary valve implantation to patients previously excluded by device size limitations.

**Figure 1:**
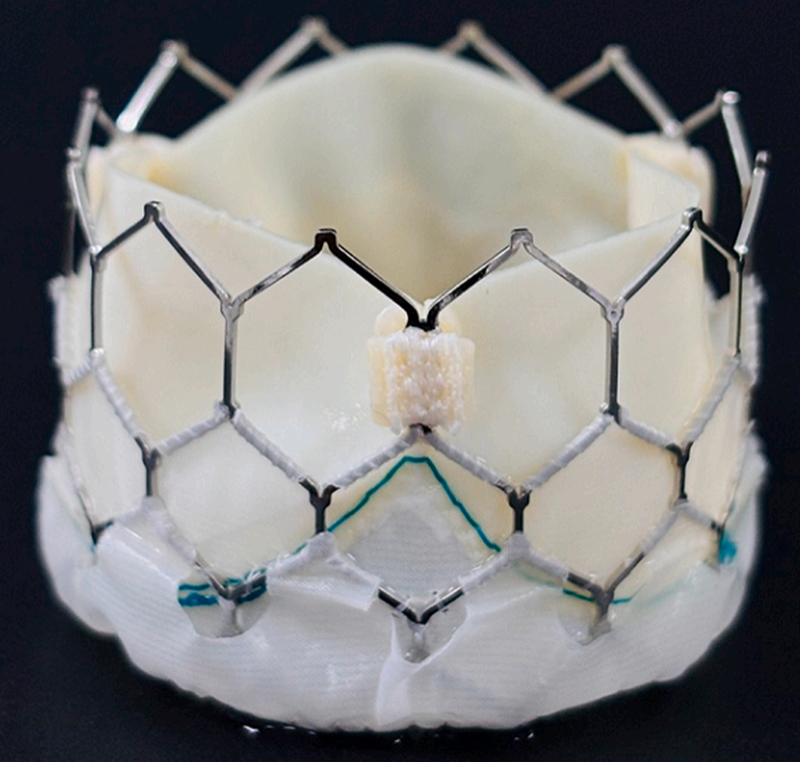
Custom 35-mm Myval Transcatheter Heart Valve Designed for Implantation in Markedly Dilated Native Right Ventricular Outflow Tracts The 35-mm Myval valve was custom-manufactured beyond the standard platform. The crimped profile measures approximately 8 mm in diameter and 31 mm in length, with a nominal balloon volume of 50 mL. In selected cases, an additional 10 mL of inflation volume was used to optimize expansion, increasing the effective diameter to ∼37.5 mm. This customization enabled transcatheter therapy in patients with extra-large native RVOTs. Note: This schematic illustration was digitally generated using AI to represent the valve platform; all content was reviewed and verified by the authors for technical accuracy. RVOT = right ventricular outflow tract.

**Figure 2:**
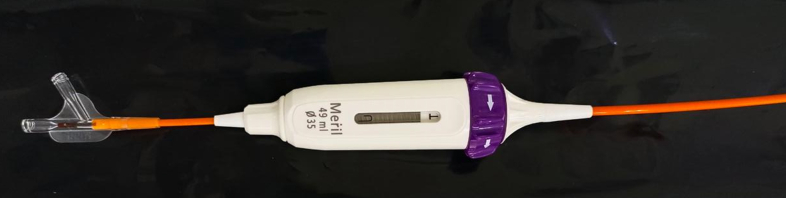
Calibrated Inflation Syringe Used for Controlled Balloon Expansion During Transcatheter Valve Implantation The device shown is a calibrated inflation syringe (Meril Life Sciences) used in conjunction with the Navigator delivery catheter for balloon-expandable valve implantation. It incorporates a rotating knob for controlled pressure delivery, a transparent chamber with a 49-mL capacity (diameter: 3.5), and a connector with flexible tubing for secure attachment. This system enables precise monitoring of inflation volume and pressure, ensuring symmetrical valve expansion and procedural safety.

The Myval transcatheter heart valve (Meril Life Sciences) is a balloon-expandable, trileaflet bovine pericardial valve mounted on a nickel-cobalt alloy frame designed for high radial strength and radiopacity (Figure 1). The valve is crimped using the Val-de-crimp tool onto the Navigator delivery catheter (Meril Life Sciences), a balloon-expandable platform that enables controlled positioning. For a 35-mm valve, the crimped profile measures approximately 8 mm in diameter and 31 mm in length, with a nominal balloon volume of 49 mL (Figure 2). The Myval transcatheter heart valve is routinely available in sizes up to 32 mm.^7^^,^^8^

In this series, a custom-manufactured 35-mm valve was used to address markedly dilated native RVOTs, thereby extending the size range beyond conventional platforms. In selected cases, an additional 10 mL of inflation volume may be used to optimize expansion, increasing the effective diameter beyond the nominal 35 mm and facilitating complete elimination of pulmonary regurgitation while maintaining symmetrical expansion.

## Case Series

### Case 1

#### Clinical timeline

A 56-year-old man with repaired tetralogy of Fallot in childhood was evaluated for progressive exertional dyspnea. Severe pulmonary regurgitation with right ventricular dilation was documented, establishing the indication for pulmonary valve replacement given symptomatic right ventricular volume overload.

#### Diagnostics

Cardiac magnetic resonance imaging (MRI) demonstrated severely dilated right ventricle (right ventricular end-diastolic volume index [RVEDVi]: 183 mL/m^2^), and severe pulmonary regurgitation (regurgitant fraction: 51%), with markedly dilated RVOT and main pulmonary artery (MPA). Computed tomography angiography (CTA) confirmed RVOT/MPA dilation up to ∼45 mm, normal coronary anatomy, and absence of compression risk. Catheterization with MPA angiography confirmed severe regurgitation and a compliant RVOT. Balloon sizing revealed a reproducible waist of 34.5 mm. Coronary testing during balloon inflation was negative. Multimodality imaging and hemodynamic measurements are summarized in Table 1.

**Table 1:** Cardiac Magnetic Resonance Imaging, Computed Tomography, Angiography, and Catheterization Measurements of Ventricular Volumes, Ejection Fractions, Pulmonary Regurgitant Fraction, and Right Ventricular Outflow Tract Dimensions

	MRI Volumes							MRI and CT Measurements				Angiography Measurements			Cardiac Catheterization Measurements	
	Right Ventricle				Left Ventricle											
	EDVi (mL/m^2^)	ESVi (mL/m^2^)	EF	PRF	EDVi (mL/m^2^)	ESVi (mL/m^2^)	EF	Pulmonary Valve Annulus (mm)	MPA (mm)	LPA (mm)	RPA (mm)	Pulmonary Valve Annulus (mm)	MPA Proximal (mm)	MPA Distal (mm)	Waist (mm)	Balloon Sizing (mm)
Case 1	183	80	57%	51%	85	37	56%	30	45	24	36	30	40	35	34.5	40/40
Case 2	148	63	62%	45%	87	56	65%	30	50	25	39	32.5	34	48	32	34 × 40
Case 3	170	73	57%	42%	78	40	53%	29	39	20	24 × 23	35	42	36	34	40/40
Case 4	161	74	55%	35%	75	45	60%	32	39	18	18	32	42	39	32	34/40

Cardiac MRI volumetry demonstrates indexed right and left ventricular volumes, ejection fractions, and pulmonary regurgitant fraction. CT and MRI provide annular and pulmonary artery dimensions, while angiography and catheterization confirm dynamic right ventricular outflow tract sizing and balloon interrogation results. This table highlights the discrepancy between static anatomical measurements and functional balloon waist dimensions guiding valve selection.

CT = computed tomography; EDVi = end-diastolic volume index; EF = ejection fraction; ESVi = end-systolic volume index; LPA = left pulmonary artery; MPA = main pulmonary artery; MRI = magnetic resonance imaging; PRF = pulmonary regurgitant fraction; RPA = right pulmonary artery.

#### Interventions

A 35-mm Myval valve was implanted directly under rapid right ventricular pacing without predilation. After deployment, significant residual valvular regurgitation was observed, and the prosthesis appeared asymmetrically expanded. Balloon postdilatation was performed, but regurgitation persisted. A second 35-mm Myval was subsequently implanted in a valve-in-valve fashion within the first prosthesis, resulting in symmetrical expansion and satisfactory valve function (Video 1).

#### Outcomes

Procedural success was achieved with a fluoroscopy time of 22 minutes. Postimplantation angiography demonstrated complete elimination of regurgitation. Echocardiography confirmed a well-seated valve with acceptable gradients (peak: ∼5 mm Hg) and no residual valvular or paravalvular regurgitation. The patient remained stable after the procedure, was discharged on lifelong aspirin and 3 months of clopidogrel, and was scheduled for follow-up echocardiography, Holter monitoring, and baseline MRI at 12 months.

### Case 2

#### Clinical timeline

A 49-year-old man with prior surgical pulmonary valvotomy and repaired partial anomalous pulmonary venous connection was evaluated for worsening pulmonary valve dysfunction and progressive symptoms.

#### Diagnostics

Cardiac MRI demonstrated a severely dilated right ventricle (RVEDVi: 148 mL/m^2^) with mild reduction in right ventricular systolic function. CTA showed a severely dilated main pulmonary artery (50 mm), a 30-mm annulus, enlarged branch pulmonary arteries, normal coronary anatomy, and no compression risk. CTA also confirmed complete interruption of the left superior pulmonary vein draining via collateral pathways. Catheterization revealed aneurysmal RVOT dilation with a narrowed segment at the pulmonary valve. Balloon sizing with a 34-mm balloon demonstrated a reproducible waist of 32 mm, and coronary testing during balloon inflation was negative. Imaging and volumetric data for this case are included in Table 1.

#### Interventions

A 35-mm Myval valve was implanted directly under rapid right ventricular pacing with 10 mL added volume. The prosthesis expanded symmetrically, resulting in complete elimination of pulmonary regurgitation (Video 2).

#### Outcomes

The procedure was uncomplicated, with a fluoroscopy time of 23 minutes. Postimplantation angiography confirmed complete elimination of regurgitation. Echocardiography demonstrated a well-seated valve with acceptable gradients (peak: ∼7 mm Hg) and no significant residual regurgitation. The right ventricle remained dilated with preserved systolic function (tricuspid annular plane systolic excursion: 21 mm). The right atrium was dilated (area: ∼28 cm^2^), with mild to moderate tricuspid regurgitation (right ventricular systolic pressure: ∼38 mm Hg). The left ventricle showed concentric hypertrophy with normal size and systolic function (left ventricular ejection fraction [LVEF]: ∼60%) and no pericardial effusion. The patient was discharged stable on lifelong aspirin and clopidogrel for 3 months, and was scheduled for routine follow-up with echocardiography and MRI.

### Case 3

#### Clinical timeline

A 17-year-old boy with congenital pulmonary stenosis, previously treated with neonatal balloon valvuloplasty, was followed up for progressive right ventricular dilation and pulmonary valve dysfunction.

#### Diagnostics

Cardiac MRI demonstrated moderate pulmonary regurgitation (regurgitant fraction: 42%) and a severely dilated right ventricle (RVEDVi: 170 mL/m^2^). CTA showed an aneurysmal MPA (39 × 38 mm), a dilated RVOT (49 × 28 mm), and enlarged branch pulmonary arteries, with normal coronary origins and no compression risk. Earlier catheterization in 2020 indicated an RVOT diameter of ∼32.5 mm, but percutaneous pulmonary valve implantation was deferred until a suitable large valve became available. At reassessment in 2025, catheterization confirmed a dilated RVOT with severe pulmonary regurgitation and a balloon waist of 34 mm. Comprehensive volumetric and multimodality measurements are presented in Table 1.

#### Interventions

Landing zone preparation was achieved with 2 overlapping 57-mm Optimus cobalt-chromium XXL 57-mm stents (AndraTec) mounted on a 40 × 40 mm Z MED percutaneous transluminal valvuloplasty catheter (NuMED). A 35-mm Myval valve was subsequently implanted, demonstrating symmetrical expansion and stable valve function (Video 3).

#### Outcomes

Postoperative echocardiography showed a well-seated bioprosthetic pulmonary valve with acceptable gradients (peak: ∼4 mm Hg), no residual valvular or paravalvular regurgitation, normal left ventricular size and systolic function (LVEF: ∼60%), and mildly dilated right ventricle with preserved systolic function (tricuspid annular plane systolic excursion: 22 mm). There were no complications. The patient recovered uneventfully, was discharged on lifelong aspirin and 3 months of clopidogrel, and was scheduled for routine follow-up, including echocardiography and MRI.

### Case 4

#### Clinical Timeline

A 28-year-old man with a history of childhood balloon pulmonary valvuloplasty was followed up for progressive pulmonary valve dysfunction and right ventricular dilation.

#### Diagnostics

Cardiac MRI demonstrated moderate pulmonary regurgitation (regurgitant fraction: ∼35%) with a severely dilated right ventricle (RVEDVi: ∼161 mL/m^2^) and preserved systolic function (right ventricular ejection fraction: 55%). CTA showed a dilated main pulmonary artery and pulmonary annulus measuring ∼32 mm, with normal coronary anatomy and no evidence of stenosis or compression risk. Catheterization confirmed mild pulmonary valve stenosis with severe pulmonary regurgitation. Balloon sizing with a 34-mm balloon demonstrated a reproducible waist of ∼32 mm, and coronary angiography during balloon inflation confirmed a safe distance from the coronaries. Multimodality imaging and catheterization data are summarized in Table 1.

#### Interventions

Landing zone preparation was achieved with two 57-mm AndraTec XXL bare stents mounted on a 40 × 4 mm Z-MED balloon. A 35-mm Myval valve with an added inflation volume of 10 mL was subsequently implanted, resulting in symmetrical expansion and complete elimination of pulmonary regurgitation (Video 4).

#### Outcomes

The procedure was uncomplicated, with a fluoroscopy time of 43 minutes. Repeated coronary angiograms confirmed unobstructed coronary flow. Postoperative echocardiography demonstrated a well-seated transcatheter pulmonary valve with normal leaflet motion, trivial residual pulmonary regurgitation, and acceptable gradients (peak: ∼7 mm Hg). The left ventricle had normal size and systolic function (LVEF: ∼60%), the right ventricle was mildly dilated with preserved systolic function, and there was mild to moderate tricuspid regurgitation (right ventricular systolic pressure: ∼37 mm Hg), with no pericardial effusion. The patient recovered uneventfully and was discharged on lifelong aspirin, 3 months of clopidogrel, and infective endocarditis prophylaxis, with follow-up echocardiography and baseline cardiac MRI planned at 6 to 12 months.

Consistent procedural success across heterogeneous anatomy supports reproducibility. Valve-in-valve strategy remains a viable bailout option.

## Discussion

This case series establishes that implantation of a 35-mm balloon-expandable Myval valve enables reproducible and safe transcatheter pulmonary valve replacement in patients with extra-large native RVOT previously considered unsuitable for TPVI. The TPVI procedure has become an established alternative to surgical pulmonary valve replacement for RVOT dysfunction, particularly in patients with repaired tetralogy of Fallot and related congenital lesions.^1^, ^2^, ^3^, ^4^ Despite consistent procedural success and favorable intermediate outcomes, applicability in markedly dilated native RVOTs has remained limited. Balloon-expandable systems such as Melody and Sapien are restricted to diameters ≤29 mm.^2^^,^^3^ In contrast, self-expanding systems such as the Venus P-valve depend on specific RVOT geometry for anchoring and may be unsuitable in compliant or aneurysmal outflow tracts.^5^^,^^6^ As a result, a significant subset of patients continues to be referred for surgical pulmonary valve replacement. Specifications of currently available extra-large valve platforms are summarized in Table 2.

**Table 2:** Comparison of Available Extra-Large Transcatheter Pulmonary Valves

Valve/Device	Valve Size (mm)	Outflow Diameter (mm)	Inflow Diameter (mm)	Waist (mm)	Length (mm)
Venus P-valve 34	34/25	44	44	34	61.5
Venus P-valve 36	36/25	46	46	36	63
Harmony valve 25	25	43	54	25	51
Alterra/Edwards	29	41	39	29	49
Myval 35	35 (expands to 37.5)	35	35	35	31

The Myval valve is routinely available up to 32 mm. In this series, a custom manufactured 35-mm valve was used, capable of expansion to approximately 37.5 mm with additional balloon volume. This extension beyond the standard platform enabled implantation in markedly dilated native right ventricular outflow tracts.

Prior evidence for balloon-expandable valves beyond 29 mm has been limited to isolated reports.^7^ In contrast, the present series provides reproducible multipatient experience, confirming feasibility across different anatomical substrates. A central observation was the discrepancy between static imaging and functional RVOT dimensions. Computed tomography frequently demonstrated marked dilation, yet balloon interrogation consistently identified a waist of approximately 32 mm, suitable for valve selection. This reinforces the principle that dynamic assessment of the landing zone, rather than maximal anatomical diameter, should guide procedural planning, consistent with prior TPVI experience.^3^^,^^4^

The technical strategy was tailored to RVOT morphology. Prestenting was employed in aneurysmal or irregular anatomies to create a stable landing zone, whereas direct implantation was feasible in focal narrowing at the native pulmonary valve. Procedural success was achieved in all cases without valve embolization, coronary compression, or significant residual regurgitation. The Myval platform provided high radial strength, controlled deployment, and the option to augment volume during inflation, facilitating precise positioning and expansion. These features distinguish balloon-expandable systems from self-expanding platforms, which rely more on native anatomy and offer less intraprocedural flexibility.

From a clinical perspective, the implications are significant. Patients with extra-large native RVOT have historically been excluded from TPVI and directed toward surgery. This series demonstrates that anatomical limitations are not absolute and that transcatheter therapy can be safely extended to this cohort. By addressing the size constraints of existing balloon-expandable platforms, these findings expand the therapeutic boundaries of TPVI and provide a viable alternative to repeat sternotomy in selected patients, particularly in resource-constrained settings or in those with multiple prior surgeries.

Several limitations must be acknowledged. The sample size is small, and follow-up is limited to early outcomes, precluding conclusions regarding long-term valve durability, right ventricular remodeling, or broader generalizability. The requirement for custom valve manufacturing may also limit immediate scalability. Nonetheless, these data provide a necessary foundation for further evaluation.

## Conclusions

This study provides the first reproducible evidence that 35-mm balloon-expandable TPVI is feasible across different anatomical substrates. By directly addressing the size limitation of existing balloon-expandable platforms, it extends the therapeutic boundaries of TPVI. It offers a complementary strategy to self-expanding valves in selected patients with large native RVOT.

### Statement of Consent

Written informed consent was obtained from all patients for publication of this case series and accompanying images.

### Ethics Statement

The study was reviewed and approved by an institutional review board (MOH/CSR/CR/26/13).

### Use of Artificial Intelligence

Artificial intelligence–assisted tools were used to support language refinement and manuscript structuring. All content was reviewed, verified, and approved by the authors, who take full responsibility for the accuracy and integrity of the work.

## Funding Support and Author Disclosures

The authors have reported that they have no relationships relevant to the contents of this paper to disclose.

## Appendix

Video 1 Direct Implantation of a 35-mm Myval Valve Performed Under Rapid Right Ventricular Pacing (Case 1) Persistent significant valvular regurgitation was noted after deployment, and the prosthesis appeared asymmetrically expanded. Balloon postdilatation did not resolve the regurgitation. A second 35-mm Myval valve was subsequently implanted within the first prosthesis in a valve-in-valve fashion, resulting in symmetrical expansion and satisfactory valve function.

Video 2 The Procedure Began With RVOT Angiography, Followed by Balloon Interrogation to Assess RVOT Diameter (Case 2) A balloon occlusion test with simultaneous coronary angiography was performed to exclude coronary compression. Direct implantation of a 35-mm Myval valve was then carried out under rapid pacing. Final angiography demonstrated stable valve position with complete elimination of pulmonary regurgitation.

Video 3 The Procedure Was Initiated With Right Ventricular and RVOT Angiography, Followed by Balloon Interrogation to Determine RVOT Diameter (Case 3) A balloon occlusion test with simultaneous coronary angiography was performed to exclude coronary compression. RVOT prestenting was then undertaken using 2 overlapping stents. Finally, a 35-mm Myval valve was implanted, with postimplantation angiography confirming stable valve position and complete elimination of pulmonary regurgitation.

Video 4 The Sequence Began With RVOT Angiography, Followed by Balloon Interrogation to Determine RVOT Diameter (Case 4) A balloon occlusion test with simultaneous coronary angiography was performed to confirm suitability and exclude coronary compression. RVOT prestenting was then undertaken using 2 overlapping stents. A 35-mm Myval valve was subsequently implanted, with postimplantation angiography demonstrating stable valve position, symmetrical expansion, and complete elimination of pulmonary regurgitation.
